# Nursing Reform

**Published:** 1907-04-27

**Authors:** 


### Nursing Administration.

### NURSING REFORM.

### IN THE HANDS OF THE TRAINING SCHOOLS.

The ultimate success of the movement in favour of nursing reform lies in the hands of the training schools. The wisdom and administrative ability which, in the course of years, has given the world the trained nurse, is still among us. It is merely a question of a few more years before the same forces will perfect the system, and will take such measures as may be necessary to prevent its deterioration at the hands of irresponsible persons. It often seems to be forgotten that all the training schools in this country, except those of the Local Government Board, are purely voluntary bodies. To attempt to legislate for them before securing their co-operation must be to court failure, and to proceed to move Parliament to legislate for the training schools without consultation with these, and against their better judgment, is but to beat the air. Whatever needs to be done in the way of reform, the schools are pre-eminently fitted to undertake for themselves, and only legislation effected through the combined action of the training schools themselves can have any hope of attaining its object. The training school is composed of three elements: the trustee, as responsible for finding the funds; the examiner, as responible for the standard of knowledge; the matron, as responsible for the standard of conduct and practical work. Unless these three elements work harmoniously in the evolution of the nurse the training school is maimed. In attempting co-operation for the purpose of legislation, and with the aim of uniformity in view, these three elements must combine, and no one of them can be omitted without danger to the best principles of training.

But the training schools are not only many in number; they are widely separated by situation, circumstances, and traditions. To attempt to ignore local traditions under circumstances affecting widespread interests is always to invite failure. It is certain that Scotch hospitals, let us say, could not cordially cohere in any scheme for nursing reform in which their only part was the selection by the Privy Council of one Scottish representative, and the election, by an irresponsible body of nurses, of a Scottish nurse and a Scottish matron who, if in actual touch with the interests they were supposed to represent, could not possibly attend regular meetings in London. The formation of Branch Councils in different parts of the kingdom would seem to be a preliminary essential to success in constituting a Board of Nursing truly representative of all the great training schools. Such Branch Councils, on which all the training schools in the particular district were strongly represented, and composed, as they would be, exclusively of men and women engaged in the actual work of training nurses, would be in the best possible position for ascertaining the right people to control nursing education. One such Council for London, two for the provinces, two for Scotland, two for Ireland, might be found sufficient, but the number would be dependent only on the extent to which different counties were prepared to combine for this purpose. The formation of such Branch Councils of nursing would greatly facilitate the election of the Central Board of Nursing, and render it entirely representative of training schools throughout the country. It would also relieve the Board of many duties which would press very heavily on any purely centralised system. It is at least highly probable that one of the first measures undertaken by a Board of Nursing under the direction of the training schools would be the regulation of examinations for nurses at the completion of their course. It is for many reasons desirable that the existing system, under which the nurse is examined in her own training school, should be continued, and the best authorities regard with considerable disfavour the proposal that examinations shall be centralised.

The appointment of examiners, and many minor matters connected with the regulation of the examinations, could more properly be left in the hands of the Branch Councils, acting always within well-defined limits, and thus matters affecting the interests of particular hospitals would have the advantage of being discussed before a committee of experts well acquainted with the local conditions, instead of before the wearied members of a Central Board at the close of a long meeting. It can hardly be too often reiterated that the success of any measure for promoting reform in nursing matters must depend entirely on the degree of spontaneous consent which it can command. The control of nursing education, conducted, as it is, for the most part in voluntary institutions, can only, with any degree of safety to the nurse and to the institution, be lodged in the hands of the training schools themselves. Every superintendent of nurses in a recognised training school ought to be drawn into direct touch with the Central Board charged with making regulations for training nurses. Under the system of Branch Councils matters lying wholly in the matron's jurisdiction would receive the attention of committees appointed from among the matrons of each district, while technical medical and surgical education would be dealt with by committees of those experienced in lecturing. Such a system would free both matrons and medical men from the questions of finance and administration which can be more conveniently undertaken by lay managers. Some such division of labour in the mass of detail which a Central Board of Nursing must face, would in all probability dissipate difficulties which at first sight may well seem insuperable.

